# Microeukaryote community coalescence strengthens community stability and elevates diversity

**DOI:** 10.1093/femsec/fiae100

**Published:** 2024-07-13

**Authors:** Máté Vass, Anna J Székely, Ulla Carlsson-Graner, Johan Wikner, Agneta Andersson

**Affiliations:** Department of Ecology and Environmental Science, Umeå University, SE-90187 Umeå, Sweden; Division of Systems and Synthetic Biology, Department of Life Sciences, Science for Life Laboratory, Chalmers University of Technology, SE-41296 Gothenburg, Sweden; Division of Microbial Ecology, Department of Aquatic Sciences and Assessment, Swedish University of Agricultural Sciences, SE-75007 Uppsala, Sweden; Department of Ecology and Environmental Science, Umeå University, SE-90187 Umeå, Sweden; Department of Ecology and Environmental Science, Umeå University, SE-90187 Umeå, Sweden; Umeå Marine Sciences Centre, Umeå University, SE-90571 Hörnefors, Sweden; Department of Ecology and Environmental Science, Umeå University, SE-90187 Umeå, Sweden; Umeå Marine Sciences Centre, Umeå University, SE-90571 Hörnefors, Sweden

**Keywords:** biotic interactions, coastal habitats, cohesion, community mixing, community stability, long-read metabarcoding

## Abstract

Mixing of entire microbial communities represents a frequent, yet understudied phenomenon. Here, we mimicked estuarine condition in a microcosm experiment by mixing a freshwater river community with a brackish sea community and assessed the effects of both environmental and community coalescences induced by varying mixing processes on microeukaryotic communities. Signs of shifted community composition of coalesced communities towards the sea parent community suggest asymmetrical community coalescence outcome, which, in addition, was generally less impacted by environmental coalescence. Community stability, inferred from community cohesion, differed among river and sea parent communities, and increased following coalescence treatments. Generally, community coalescence increased alpha diversity and promoted competition from the introduction (or emergence) of additional (or rare) species. These competitive interactions in turn had community stabilizing effect as evidenced by the increased proportion of negative cohesion. The fate of microeukaryotes was influenced by mixing ratios and frequencies (i.e. one-time versus repeated coalescence). Namely, diatoms were negatively impacted by coalescence, while fungi, ciliates, and cercozoans were promoted to varying extents, depending on the mixing ratios of the parent communities. Our study suggests that the predictability of coalescence outcomes was greater when the sea parent community dominated the final community, and this predictability was further enhanced when communities collided repeatedly.

## Teaser

Mixing freshwater and brackish sea communities in a small-scale experiment demonstrated increased diversity and stability, driven by competitive interactions, with the dominance of the more stable sea community enhancing the coalescence predictability.

## Introduction

Community coalescence is a complex phenomenon that involves the mixing of microbial communities from previously isolated environments (Rillig et al. 2015). Such mixing events comprise the movement and potential mixing of environments, resulting in environmental coalescence, as well as the dispersal of microbial communities termed biotic coalescence. Coalescence events are expected to impose dual impacts on communities in the form of environmental filtering due to the altered environment following mixing, and community reorganization by the dynamic rearrangement of ecological interactions, covering competitive/facilitative interactions, species corecruitment, and trophic interactions (Rillig et al. 2015, Custer et al. 2023). Interestingly, colliding communities maintain some of these biotic interactions as intact, thus stabilizing the coalesced community (also known as network coherence) (Rillig et al. 2015). Hence, the likelihood of community establishment following coalescence can depend on biotic interaction types and the cohesiveness of taxa within the parent communities. The recently developed ‘cohesion’ metric (Herren and McMahon 2017) quantifies the degree to which members of a community are connected and can be utilized to evaluate its impact on community stability (see e.g. Hernandez et al. 2021). Higher fraction of positive cohesion indicates greater environmental synchrony and/or facilitative interactions between taxa, which in turn results in a community with the potential for mutual downfall (Coyte et al. 2015). This happens, for example, when the decreasing abundance of one species pulls others down and by doing so, destabilizes the community via positive-feedback loops. In contrast, communities with a greater fraction of negative cohesion, attributed to competition and/or environmental filtering, tend to be more stable (e.g. have low species turnover over time) due to dampened positive feedback loops and reduced dependency (coupling) between species (Herren and McMahon 2017, Bier et al. 2022). Inferring community stability from community coherence, thus, represents a promising avenue for understanding and predicting the outcome of community coalescence. Previous works suggest that parent communities with more facilitative interactions contribute to a greater proportion of species in the final coalesced community due to their superior ability to deplete resources and resist invasions (Chang et al. 2021, Lechón-Alonso et al. 2021), especially when coalescence happens repeatedly (Lechón-Alonso et al. 2021, Song et al. 2021). Thus, the temporal scale on which coalescence events occur, for example, the frequency of invasion events (i.e. one-time versus repeated coalescence) have most likely plays a key role in determining the dominance of interaction type of a community network, and consequently, coalescence outcomes.

Coalescence events are particularly common in aquatic ecosystems as water bodies of different origins often mix at interfaces like river–sea junctions (Rillig and Mansour 2017). Such estuary habitats present challenging environments for microbial communities in respect of salinity, oxygen levels, and nutrient concentrations, which vary greatly not only spatially along the mixing zones but also temporarily as a result of the fluctuations that emerge due to hydrological features of river inflows and tidal intrusions (Wolanski et al. 2012, Lee et al. 2017, Mansour et al. 2018). This spatio-temporal environmental variability drives the development of diverse microbial communities, often characterized by protistan species maxima (Telesh et al. 2011). In estuaries, communities continuously coalesce and form a new set of populations with different community structure and stability. Only microbes that are able to adjust their osmoregulation and metabolic profiles (i.e. nutrient acquisition) and/or elevate their growth rates can survive these rapidly changing conditions (Bouvier and del Giorgio 2002, Balzano et al. 2015, Tee et al. 2021). Such river–sea mixing events are particularly common in the Baltic Sea, which is a shallow brackish sea characterized by large river influence (Raudsepp et al. 2023) making it a perfect environment to study the process of whole-community mixing. Past works suggest that even low salinity environments, such as the Baltic Sea, impact river-transported microbes lacking adaptability to saline conditions (Langenheder et al. 2003, Shen et al. 2018), shifting the final, mixed community towards that of the sea (Székely et al. 2013, Rocca et al. 2020, Song et al. 2022).

Although microeukaryotes play crucial roles in aquatic primary production and nutrient cycling via their roles in the food web, only a few studies have investigated the eukaryotic fraction of the microbial consortia along river-to-sea transects (Tee et al. 2021, Yang et al. 2021, Vass et al. 2022). Community coalescence and the mechanisms underlying its outcomes are nevertheless scarcely studied along river to sea transitions (Mansour et al. 2018). Therefore, we aimed to mimic estuarine condition in a microcosm experiment by mixing freshwater river community with brackish sea community and specifically investigate whether the fate of microeukaryotes during community coalescence differ in response to varying mixing frequencies and ratios.

Here, we address two fundamental questions about community coalescence: (i) Does mixing ratio define the outcomes of community coalescence? (ii) What effect does mixing frequency (one-time versus repeated coalescence) have on the final community composition?

Beginning with two microbial communities originating from a river and an offshore site in the Gulf of Bothnia, we inoculated each of them separately in their mixed environment to assess environmental coalescence. Community coalescence outcomes were also assessed after mixing them one-time or repeatedly at three different mixing ratios, varying the initial ratio of the parent communities. We hypothesized that (a) it is possible to predict the outcome of community coalescence based on the applied mixing ratios and the individual environmental adaptive capabilities of the parent communities, and further, (b) that one-time coalescence is advantageous for competition-driven (stable) communities, while repeated mixing of communities eventually results in facilitation-dominated communities, as suggested by Lechón-Alonso et al. (2021).

## Material and methods

### Sampling

The two microbiomes for our microcosm experiment were collected on 25 April 2022, from a subarctic coastal area of the Gulf of Bothnia, Sweden, which coincided with the diatom spring bloom. The freshwater river sample (*in situ* temperature: 2.8°C, pH: 5.6, salinity: 0.1 psu) originated from a coastal river, Ängerån (63°34′51.8ʹʹ N; 19°50′07.0ʹʹ E). This river has a moderate ecological status and not heavily affected by anthropogenic influences, according to the Water Information System Sweden (viss.lansstyrelsen.se). The brackish seawater (*in situ* temperature: 5.2°C, pH: 7.5, salinity: ∼4 psu) was collected from an offshore site (63°28′30.20″ N; 19°50′5.85″ E), ∼12 km from the mouth of the river Ängerån. River and sea samples were taken from the euphotic zone (integrated sample from 0 to 10 m depth, in the case of sea water) and transported to the laboratory in sterile containers. The samples were prefiltered through a 200-µm mesh to remove macroorganisms (i.e. mesozooplankton) and debris, and used immediately to set up the experiment. Total dissolved nitrogen (TDN) and phosphorus (TDP) were also measured, following standard analytical methods described in Hansen and Koroleff (1999).

### Coalescence experiment

**Figure 1.**
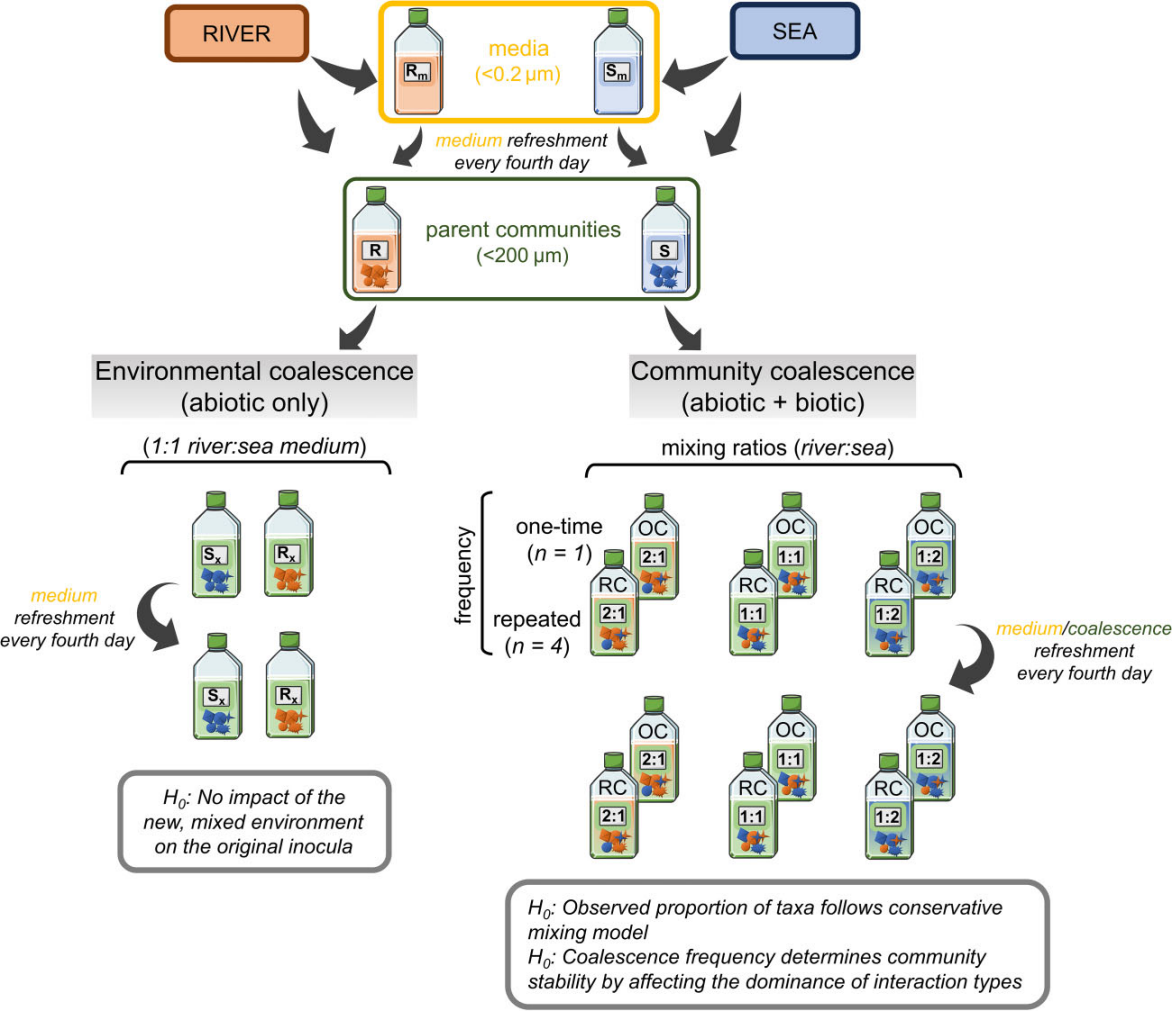
Overview of the experimental design. Prefiltered (<200 µm) water samples from two sites (river and sea) were collected to inoculate our microcosms. Environmental coalescence: each parent community **(R, S)** was used to assess the potential environmental filtering effect of the mixed medium (river:sea = 1:1) on the unmixed parent communities (R_x_ and S_x_). Community coalescence: two different coalescence treatments (one-time—OC, and repeated—RC) were set up as follows. River and sea parent communities were mixed at three mixing ratios (1:1, 1:2, and 2:1, respectively) to create coalesced cultures. For the cultures exposed to environmental coalescence and the ones undergoing OC treatment, sterile-filtered (<0.2 µm) media (R_m_ and S_m_) were used during the course of the experiment (i.e. every fourth day) to refresh the parent communities. Cultures exposed to repeated coalescence (RC) received both parent communities mixed according to the corresponding coalescence treatment instead of the filtered media. All experimental treatments (each of 60 ml) consisted of five replicates. Parts of the figure were drawn by using pictures from Servier Medical Art. Servier Medical Art by Servier is licensed under a Creative Commons Attribution 3.0 Unported License (https://creativecommons.org/licenses/by/3.0/).

A 16-day long experiment was conducted, using parent communities prepared from the river (R) and sea (S) samples (Fig. 1). These parent communities (R and S) were then exposed to one-time (OC) or repeated (RC) coalescences in three river:sea mixing ratios (1:1, 1:2, and 2:1).

To avoid substantial chemical changes due to autoclaving, river (R_m_) and sea (S_m_) media was prepared by sequentially filtration through GF/F filters (0.7 µm, Whatman) and then through sterile 0.2 µm 47 mm membrane filters (Pall Supor) in a laminar flow hood. Although this serial filtration was sufficient for elimination eukaryotic cells, prokaryotic cells were not completely removed and thus, axenic conditions were not achieved. However, only the sea medium experienced increased bacterial abundance by the end of our experiment (i.e. day 16; Supplementary Fig. S1).

To assess the individual environmental adaptive capabilities of the parent communities and estimate the effect of coalescence imposed solely by the abiotic environmental changes of the mixed media (i.e. environmental coalescence), river and sea inoculum (80%; v/v) were incubated separately in blended native media (1:1 mixture of R_m_ + S_m_), hereafter R_x_ and S_x_ (i.e. environmental coalescence), respectively. For the community coalescence treatments, the mixture of R_m_ and S_m_ were inoculated with river (R) and sea (S) parent communities (80%; v/v), according to the applied mixing ratios (e.g. OC_1:2_/RC_1:2_ treatment consisted of 4 ml R_m_ + 8 ml S_m_ and 16 ml R + 32 ml S) in order to achieve equally dominated, sea-dominated or river-dominated conditions.

Every 4 days 20% sample volume of each OC microcosm was exchanged with the respective medium, following the initial mixing ratios. For this, each replicate ‘A’ of the communities received medium from replicate ‘A’. Likewise, each replicate ‘B’ received medium from replicate ‘B’, and so on. Microcosms of RC treatment was exchanged with samples from the respective inoculum communities, instead of the filtered media, applying 20% (v/v) exchange. Both the community coalescence and the medium replacement were carried out in a laminar flow hood, using sterile disposable pipettes.

All treatments (60 ml each) with five replicates were maintained in sterile culture flasks with filter caps (Sarstedt, Nümbrecht, Germany), resulting in 60 cultures in total. The incubation was carried out at 10°C with a photoperiod set to 17:7 h light:dark cycle to mimic ambient conditions (Andersson et al. 1994). Twice a day, the microcosms were mixed by gently shaking and randomly placed to minimize the potential biases of the differential light in the experiment room.

### Monitoring microcosms during the experiment

Every 4 days subsamples (12 ml) from each culture flask were pipetted into sterile 15 ml tubes before the exchange of medium and/or parent community and processed as follows.

Bacterial abundances of glutaraldehyde-fixed samples (1 ml with 1% final concentration) were determined by flowcytometry (BD FACSVerse instrument, BD Biosciences) using SYBR Green I (Invitrogen) staining dye (Marie et al. 1997). To assess and compare the growth of algae across microcosms, the subsamples were dark-adapted for at least 20 min and chlorophyll fluorescence-induced dynamic curve was measured using AquaPen-C device (Photon Systems Instruments, Brno, Czechia). The strong correlation between the integral area of chlorophyll fluorescence induction (OJIP) curve and chlorophyll-a content allowed us to estimate chlorophyll content of samples in a quick, noninvasive way (Chen et al. 2021). The validity of this method was checked by measuring the ethanol-extracted (95%) chlorophyll-a concentration of the initial water samples using spectrofluorometer and correlated it with the integral area of the measured OJIP curves (*R*^2^ = 0.91). We acknowledge that this method has limitations, nevertheless, can be used to monitor our microcosms and to compare them among treatments.

At the end of the experiment (day 16) subsamples were filtered through 0.2-µm syringe filters and kept frozen until the measurement of chemical properties (e.g. total dissolved nutrients). TDN and TDP, were measured, following standard analytical methods described in Hansen and Koroleff (1999). The remaining sample volumes were filtered by vacuum filtration onto 0.2 µm 47 mm membrane filters (Pall Supor) and the filters were stored at –80°C.

### Community analysis by long-read amplicon sequencing

The DNA was extracted from the filters using the ZymoBIOMICS DNA Miniprep Kit (Zymo Research Corp, CA, USA) following manufacturer’s protocol. DNA extracts were quantified with NanoDrop (ND-1000 Spectrophotometer).

Amplification was done using the V4_Balzano_F/D11_3143R primer pair (see, Supplementary Table S1) in order to amplify almost the whole (∼4.5 kb) eukaryotic rRNA operon (Latz et al. 2022), which allows better taxonomic classification. The PCR was performed according to Latz et al. (2022), using 20 ng template DNA. The barcoded PCR products were purified with 0.8× of AMPure magnetic beads (Beckmann) following the manufacturer’s protocol. Thereafter, the purified PCR products were quantified using the Qubit 1× HS Assay Kit (ThermoFisher Scientific) and pooled in equimolar amounts.

A total of 1 µg of library was used for the ONT library preparation using the 1D sequencing (SQK-LSK109; Oxford Nanopore Technologies), following some modifications described in Vass et al. (2022). Sequencing was performed using a MinION Mk1C instrument (ONT) operated with a Spot-ON Flow Cell (R9.4.1 chemistry). Real-time high-accuracy basecalling (HAC) was executed using the MinKNOW software (v22.05.6), resulting in 2.43 M reads Q > 9.

Quality reads were demultiplexed and barcoded primers were trimmed with MiniBar (Krehenwinkel et al. 2019), filtered by length (2–6 kb) with NanoFilt (v2.8.0) (De Coster et al. 2018), and processed using the NGSpeciesID pipeline (v0.1.2.2) (Sahlin et al. 2021, Pomerantz et al. 2022) with –mapped_threshold 0.8 –aligned_threshold 0.6 parameters during read clustering by isONclust (Sahlin and Medvedev 2020). We obtained on average 29 078 reads per sample with 62.1% mapping rate (i.e. % of high-quality paired reads for generation of the consensus sequences from total reads) (Supplementary Table S2). Quality-filtered (Q > 9 and 2–6 kb) reads were deposited to NCBI SRA database under the accession number PRJNA922225.

18S, 28S rRNA genes (SSU and LSU) and the full length internal transcribed spacer (ITS) were extracted using ITSx (Bengtsson-Palme et al. 2013) and used in BLASTn search to assign taxonomy against the PR2 v4.14 database (Guillou et al. 2013), SILVA LSU v138.1 reference database (Quast et al. 2012) and the UNITE+INSD v9.0 database (Abarenkov et al. 2022), respectively, using BLAST+ (v2.11.0+) and keeping hits with at least 80% identity. Results of the BLASTn search were processed with phyloR (https://github.com/cparsania/phyloR) to keep top hits and to assign taxonomy levels. Each operational taxonomic unit (OTU) was manually inspected by determining consensus classification down only to the level that could be robustly supported by at least two of the three reference databases, using the 2 out of 3 rule (e.g. if an OTU classified as taxon A by two reference databases but as taxon B by the third one, then taxon A is selected). Noneukaryotic consensus sequences and their corresponding OTUs were discarded from the OTU table (*n* = 130). The taxonomic distribution of reads was visualized with Krona (http://sourceforge.net/projects/krona).

### Data analysis

All statistical analyses and visualizations were conducted in R version 4.0.4 (R Core Team 2021). Rarefaction was done using 4752 reads per sample, resulting in 461 OTUs. The final OTU table and the corresponding taxonomy are available in Open Science Framework (https://osf.io/sme36). Sample coverage was assessed with the ‘iNext’ R package (Hsieh et al. 2016), and found that community composition was sufficiently covered (Supplementary Fig. S2). Differences in total dissolved nutrients (i.e. TDN and TDP) across inoculum sources and treatments were assessed by pairwise Wilcoxon rank-sum test with a Benjamini–Hochberg (BH) corrected significance cutoff of 0.05. Diversity analyses (alpha-diversity and beta-diversity based on Bray–Curtis distance) were performed using the ‘microeco’ R package (v.0.6.5) (Liu et al. 2021) and the results (i.e. nonmetric multidimensional scaling—NMDS) were plotted using ‘ggplot’ package (Wickham 2009). Difference in alpha diversity across inoculum sources and treatments were tested with ANOVA followed by Duncan's test (*P* < .05) as a *post hoc* test. To test compositional differences between samples, pairwise permutational multivariate analysis of variance (PERMANOVA, permutations: 999) was performed using the function pairwise.adonis in ‘pairwiseAdonis’ R package (Arbizu 2019).

#### Community stability inferred from community cohesion

To estimate network coherence that impacts community stability, we first quantified the abundance-weighted pairwise correlations of every OTU and used the resulting positive and negative co-occurrences separately to calculate negative and positive community cohesion, respectively, as proposed by Herren and McMahon (2017). Cohesion is a metric that measures the degree of connectivity of each observed microbial community. Throughout this paper, we infer community stability from the absolute value of the ratio of negative and positive cohesion (negative: positive), as in Hernandez et al. (2021). This community stability metric takes < |1| values when communities have higher proportions of facilitation than competition, while values > |1| suggest competition-dominated communities, and thus, a community with more negative-feedback loops. Through such negative-feedback loops propagation of perturbations to the rest of the community is dampened, leading to greater overall community stability (Fontaine et al. 2011, Coyte et al. 2015).

Note that for the estimation of community stability, we used nonrarefied dataset as suggested by Herren and McMahon (2017). Differences in community stability across inoculum sources and treatments were assessed by pairwise Wilcoxon rank-sum test with a BH corrected significance cutoff of 0.05.

#### Evaluation of the environmental and biotic component of community coalescence

Changes in the relative abundance of each OTU in the parent inoculum communities (i.e. R or S) compared to their abundance in mixed media of the environmental coalescence treatment (R_x_ or S_x_) were assessed by differential abundance analysis using ZicoSeq (permutation: 999) (Yang and Chen 2022). Only taxa that were present in both the parent community (i.e, R or S) and the corresponding environmental coalescence treatments (i.e. R_x_ or S_x_), and that were not affected by the effect of environmental coalescence (i.e. showed no significant (*p*_FDR.adj_ < 0.05) decrease in abundance in R_x_/S_x_ compared to R/S) were selected for the subsequent analyses. This ensured to filter out taxa affected by environmental coalescence (i.e. due to environmental filtering) and allowed us to assess the population-level dynamics attributed to the biotic component of community coalescence.

To evaluate the outcome of such community coalescence (i.e. biotic component of community coalescence), we quantified the extent of deviation between the observed coalesced communities and those expected according to a conservative mixing model as in Székely and Langenheder (2017) and Vass et al. (2021). For the expected communities we used the calculated OTU proportions from the two parent communities (R and S) with the applied mixing ratios and compared them with the observed OTU table (for detailed calculations consult Supplementary data Equation S1).

Thereafter, we calculated the Bray–Curtis similarities of the observed and expected coalesced communities. The comparison of the Bray–Curtis similarities was used to indicate the predictability of coalescence outcomes and tested using *t*-tests. Specifically, no significant deviation (*P* < .05) indicates that the observed community does not differ significantly from the expected one, suggesting predictable community coalescence. Differences between community coalescence treatments were assessed using a one-way ANOVA and a subsequent Tukey’s HSD test. Additionally, Bray–Curtis similarities between coalesced communities (i.e. OC and RC) and the corresponding parent communities (i.e. R and S) for the observed and expected data matrices were also calculated, separately. Here, a significantly greater deviation between observed versus expected similarity (*P* < .05) indicates that coalescence resulted in greater community divergence from the parent communities than expected. On the other hand, a significantly lower deviation (*P* < .05) indicates a higher convergence towards the parent communities than expected, which could be a consequence of asymmetric coalescence outcome (i.e. the dominance of one parent community in the final, coalesced community).

To assess population dynamics in response to community coalescence, we used further differential abundance analyses (ZicoSeq; permutation: 999) to detect OTUs with significant (*p*_FDR.adj_ < 0.05) increase or decrease in taxa abundances in the coalesced communities compared to their abundances in the parent communities (R, S). Finally, Kruskal–Wallis test (since the assumptions of two-way ANOVA were not met) were applied to reveal whether the different coalescence treatments, or mixing ratios, resulted in different total relative abundance of OTUs that increased or decreased after community coalescence.

## Results

### Environmental condition of microcosms

Our parent (R, S) and coalesced communities (OC_1:1/1 : 2/2 : 1_ and RC_1:1/1 : 2/2 : 1_) showed distinct chemical and compositional properties. Total dissolved nutrients (i.e. TDN and TDP), as well as chlorophyll-a concentration—as a proxy of the biomass of primary producers—showed variation across microcosms (Supplementary Figs S3 and S4). On average, sea medium (S_m_) was nitrogen-poor (75.75 µg/l) compared to the river medium (R_m_: 451.24 µg/l) (Kruskal–Wallis: *p_adj_* < 0.05), and both had low levels of dissolved phosphorus (TDP; S_m_: 2.94 µg/l, R_m_: 3.2 µg/l). Microcosms exposed to environmental coalescence (i.e. R_x_, S_x_) did not suggest nutrient-poor conditions by the end of the experiment, as they showed similar TDP (3.36–3.72 µg/l) and greater TDN (227.34–342.98 µg/l) values than those observed in filtered media (S_m_, R_m_) (Supplementary Fig. S3). Cultures of community coalescence treatments had even greater (Kruskal–Wallis: *p_adj_* < 0.05) availability of TDP (4.98 µg/l) than all the other microcosms (except sea inoculum), and their TDN levels (292.69 µg/l, on average) were intermediate between the levels of R_m_ and S_m_, showing significant differences (Kruskal–Wallis: *p_adj_* < 0.05) in relation to the applied mixing ratios. The total dissolved nutrients, however, did not differ between one-time and repeated coalescence treatments.

Our inocula originated from oligotrophic ecosystems, hence, the overall observed low values of chlorophyll-a (0–4 µg/l) are not peculiar. Estimated biomass of primary producers was significantly higher (Tukey’s HSD: *P* < .001) in sea (S: 3.12 µg/l) than in river (R: 0.78 µg/l) parent communities (Supplementary Fig. S4). By the end of the experiment (i.e. day 16), these parent communities also significantly differed (Tukey's HSD: *P* < .001) from their respective communities that have been exposed to mixed media (S versus S_x_ and R versus R_x_). Interestingly, microcosms with sea inoculum reached, on average, higher chlorophyll-a levels when incubated in the mixed (S_x_: 2.66 µg/l) than in their original medium (S: 1.62 µg/l). In contrast, river community grew better in their original medium (R: 1.08 µg/l) compared to the mixed environment (R_x_: 0.32 µg/l). Biomass values in both coalescence frequency treatments converged by day 16 (Tukey’s HSD: *P* > .05), despite their initial differences (i.e. day 4) (Tukey’s HSD: *P* < .001; except between OC_1:1_ and OC_2:1_ communities) (Supplementary Fig. S4). Here, we also found that algal biomass decreased over time in microcosms with greater sea microbiome dominance (i.e. mixing ratio of 1:2), in contrast to river inoculum-dominated microcosms wherein the biomass showed an increased trend.

### Community diversity

Out of the identified 461 microeukaryotic OTUs, 84 were shared among all microcosms (Supplementary Fig. S5). Communities with only sea inoculum (e.g. S and S_x_) had the least numbers of unique OTUs (S = 5, S_x_ = 2). Interestingly, communities exposed to community coalescence harboured the most unique taxa (OC: 15, RC: 23), comprising mostly of fungal taxa. Taxa richness and Shannon’s diversity suggested elevated alpha diversity of microcosms exposed to repeated coalescence (Duncan’s multiple range test for ANOVA: *P* < .05), and no significant differences between inoculum sources (except the inverse Simpson's index) (Supplementary Fig. S6). Treatments with repeated coalescence and river dominance (e.g. RC_2:1_) had the highest alpha diversity estimates (richness: 219–229, Shannon: 4.33–4.53, and inverse Simpson: 42.53–61.75).

### Community structure and stability

**Figure 2.**
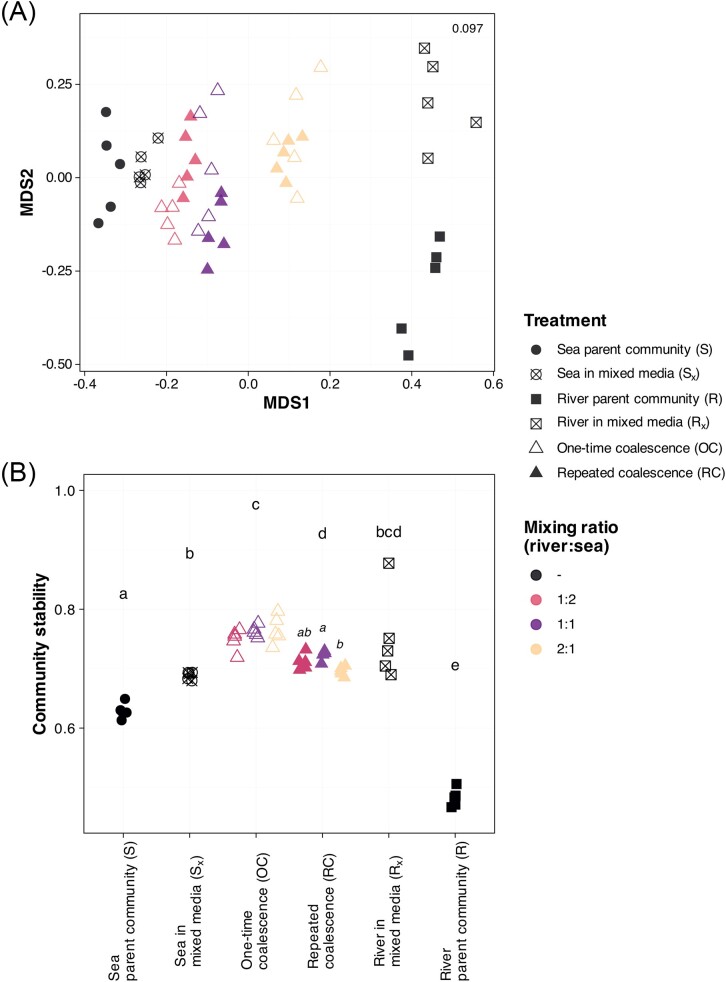
(A) Microeukaryotic community compositions across parent communities and coalescence treatments. Stress value is shown on the upper right corner. (B) Community stabilities based on the ratio of negative:positive cohesion (Herren and McMahon 2017) across parent communities and treatments. Significant (*P* < .05) differences in community stability across samples and mixing ratios are represented by lowercase and italicized letters, respectively. *N* = 5 for each type of sample and treatment. Error bars indicate standard deviations.

The NMDS of the microeukaryotic communities (Fig. 2A) together with the pairwise PERMANOVA results showed that the parent communities (S and R) and those exposed to environmental coalescence (S_x_ and R_x_), by mixing media with 1:1 ratio, compositionally differed (pairwise PERMANOVA, *P* < .05), indicating the impact of environmental filtering. Communities exposed to different community coalescence treatments were also significantly different from each other (pairwise PERMANOVA, *P* < .05), except for OC_1:1_ and RC_1:1_, as well as OC_1:1_ and RC_1:2_. Although complete convergence to each of the parent community did not occur in any of these communities, the compositions of all community coalesced treatments shifted towards sea parent community (S).

When we inferred community stability from the ratio of negative versus positive community cohesion, we found that communities had high proportions of positive cohesion (attributed to facilitative interactions) in all cases (ratio of negative: positive cohesion < |1|), indicating numerous positive-feedback loops that lead to low stability (Herren and McMahon 2017, Hernandez et al. 2021). Nevertheless, sea parent communities (S) were significantly more stable (BH-corrected Wilcoxon test: *P* < .05) than river parent communities (R) (Fig. 2B), and S community stability increased when they were grown in mixed media (S_x_ and R_x_). Similarly, coalesced communities, OC treatments in particular, had greater community stability (i.e. increased proportion of negative cohesion) than their respective parent communities. Differences in mixing ratios had only an effect in the case of repeated coalescence treatments (Fig. 2B), wherein community stability presented significant decreasing difference (BH-corrected Wilcoxon test: *P* < .05) between RC_1:1_ and RC_2:1_.

### Compositional dynamics imposed by environmental and community coalescence

From a compositional point of view, sea inoculum represented a diatom and dinoflagellate-dominated community (Supplementary Fig. S7), while river inoculum was dominated by golden algae (e.g. Crysophyceae) and ciliates (Supplementary Fig. S8). Differential abundance analysis revealed ten OTUs with >10% prevalence (i.e. OTUs present in more than 10% of the samples) in the sea inoculum (mainly Ochrophyta and Dinoflagellata) and forty in the river inoculum (mainly Ochrophyta). These OTUs were negatively affected (*p*_FDR.adj_ < 0.05) by environmental coalescence (in S_x_ and R_x_) (Supplementary Figs S9 and S10).

**Figure 3.**
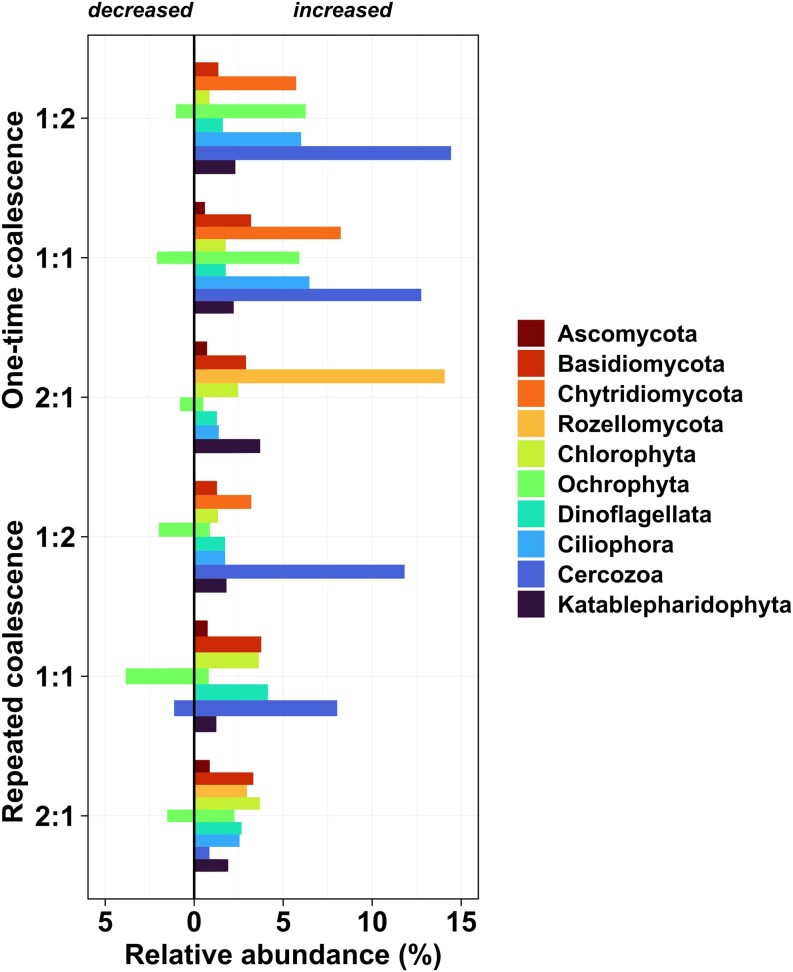
Relative abundances (>0.5%) of microeukaryotes in the coalesced communities across different community coalescence treatments with three mixing ratios (river:sea). OTUs of coalesced communities showing significant (*p*_FDR.adj_ < 0.05) increase or decrease in taxa abundance due to biotic component of community coalescence, compared to their parent communities, were identified by differential abundance analysis, and grouped by higher taxonomic levels for clarity.

To assess the pure effect of biotic component of community coalescence in the subsequent analyses, we filtered out taxa that had been negatively impacted by environmental coalescence. Thereafter, our differential abundance analysis revealed numerous OTUs (prevalence > 10%) that decreased or increased in abundance. The relative abundances of these differentially abundant taxonomic groups are presented in Fig. 3. We found that mainly diatoms (e.g. *Chaetoceros, Thalassiosira*, and *Skeletonema*) within the Ochrophyta phylum decreased in abundance (by 1.2% on average) after community coalescence (Fig. 3). In contrast, community mixing resulted in increased abundances of numerous microeukaryotes including fungi (with an increase of 3.3% on average, e.g. Ascomycota, Basidiomycota, and early-diverging zoosporic fungi), ciliates (+2.8%) and other microeukaryotes [i.e. Cercozoa (+8.4%) and Katablepharidophyta (1.9%)] (Fig. 3). There were general trends showing that the relative abundance of Cercozoa was higher (+3.5% on average) in the final (i.e. day 16) coalesced communities with more sea inoculum (i.e. mixing ratio of 1:2), while Ascomycota, Basidiomycota, Rozellomycota, and Chlorophyta OTUs had greater relative abundances (+2.1% on average) in river dominated coalesced communities (i.e. mixing ratio of 2:1).

The total relative abundances of the significantly decreased OTUs (selected based on the differential analysis) were significantly higher in repeated versus one-time coalescence treatments (χ^2^ = 23.77, *P* < .001). In contrast, coalescence frequency (one-time versus repeated) had no effect on the relative abundance of OTUs (χ^2^ = 2.19, *P* = .139) which maintained or significantly increased (*p*_FDR.adj_ < 0.05), following mixing.

### Predictability of the biotic component of coalescence outcomes

**Figure 4.**
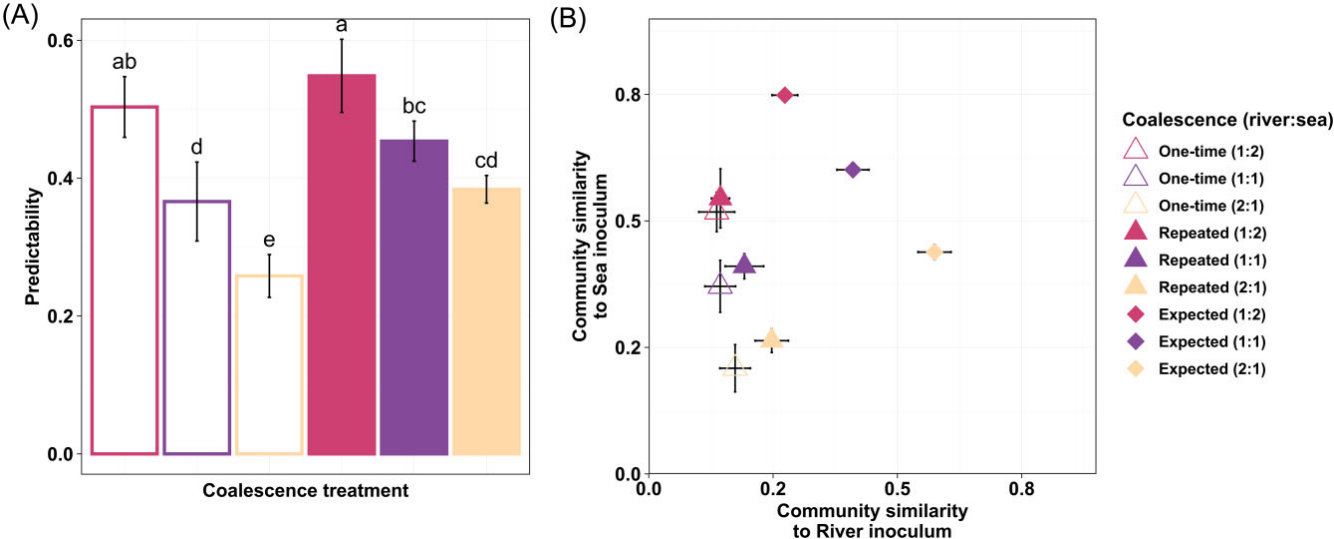
(A) Predictability of biotic component of community coalescence outcomes increases with the ratio of sea community in the final coalesced communities based on the similarity of the observed and expected communities. All observed communities significantly differ from the expected communities (*P* < .001). Significant (*P* < .05) differences among treatments are represented by lowercase letters. (B) Similarity of the observed and expected coalesced communities in relation to their parent communities. Expected community similarities were determined by conservative mixing model based on the applied mixing ratio or river and sea parent community (see the section ‘Methods’ for details). *N* = 5 for each type of treatment. Error bars indicate standard deviations.

The observed coalesced communities differed in all cases from their corresponding expected community compositions (*t*-test: *P* < .001) and their predictability differed across coalescence treatments (ANOVA: F = 32.23, *P* < .0001) (Fig. 4A). A clear pattern suggesting decreasing predictability of the coalesced communities with increasing ratio of river inoculum (i.e. predictability of river: sea mixing: 2:1 < 1:1 < 1:2) was found (Fig. 4A). In addition, predictability was typically lower in communities exposed to one-time coalescence than in those subjected to repeated coalescence (*P* < .05).

The observed Bray–Curtis similarity between each coalesced community and its parent community was calculated and compared to the expected similarity (Fig. 4B). This showed that all communities had lower similarity to their parent communities than expected (ANOVA: *F* = 69.12, *P* < .001) (Fig. 4B). Furthermore, communities that were either exposed to one-time coalescence treatment (i.e. empty triangles) or received higher proportion of river inoculum (i.e. yellow triangles) tended to diverge even more from the expected similarities than those exposed to repeated coalescence treatments and having lower ratio of river inoculum, respectively. On average, coalesced communities showed greater similarity to sea (β_Bray–Curtis_: 0.39) than to river (β_Bray–Curtis_: 0.17) parent communities (Fig. 4B).

## Discussion

In this study, we mimicked estuarine conditions by mixing a subarctic freshwater river community with a brackish water community from the Gulf of Bothnia and assessed the outcome of different mixing scenarios. We observed asymmetrical community coalescence outcomes as coalesced communities were generally shifted towards the sea parent community, which was also generally less impacted by the effect of environmental coalescence (i.e., environmental filtering). Community coalescence increased community stability and most likely promoted competitive interactions with the introduced species, leading to a stabilizing effect by negative-feedback loops. Overall, the predictability of coalescence outcomes was greater when sea microbes dominated the final community, and this predictability increased when communities were repeatedly mixed.

### Compositional dynamics imposed by coalescences

The sampled water bodies are characterized by oligotrophic conditions (Andersson et al. 1996, Wasmund et al. 2001, Wikner and Andersson 2012). Specifically, our inocula originated from phosphorus- and nitrogen-limited river and sea habitats, respectively. In such oligotrophic environments, we expect species to be under higher stress than in nutrient-rich environments (Ornolfsdottir 2004).

River communities subjected to environmental coalescence suffered a four times greater taxa loss (8% of riverine OTUs), compared to the sea microbiome (2% of marine OTUs). This suggests, in line with Cloern et al. (2017) and Rocca et al. (2020), that sea microeukaryotes are better adapted to the new environment imposed by habitat mixing, probably due to their brackish origin, making them more tolerant to saline conditions than freshwater species. However, most diatoms, the group that suffered the most from the biotic effects of community coalescence, originated from sea communities.

In community coalescence treatments, unequal mixing ratios of river and sea communities resulted in contrasting algal biomass, wherein primary producers decreased in sea-dominated coalesced communities, while increased in river-dominated microcosms over time. A possible explanation for this phenomenon is that the more saline mixed medium causes riverine algal biomass to decline (i.e. filtered by the environment), which leads to the opening of niches that the more salt tolerant sea algae can occupy and utilize the river-derived high nitrogen supply for their growth. Nevertheless, it seems that changes in water conditions exert minimal influence on microeukaryotes in general, as evidenced by the low percentages of species loss during environmental coalescences. This might be the consequence of the small changes in salinity being within the tolerance range of microeukaryotes, or due to complex ecological interactions where changes in salinity due to habitat mixing influence heterotrophic microeukaryotes. For example, the decline of certain microbes, as discussed above, opens niches and releases organic matter for the microbial loop, supporting bacterial growth and thereby bacterivorous microeukaryotes (Stefanidou et al. 2018). We can speculate that this process can further be promoted by the elevated photosynthesis (observed in river-dominated coalesced communities, see e.g. Supplementary Fig. S4) that might have increased pH (not measured herein) and in turn released coprecipitated P into the water, as evidenced by the increased TDP levels in coalesced communities. In addition to environmental filtering, enhanced biotic interactions (e.g. diatom—chytrids) (Vass et al. 2022), as well as the unexpected bacterial growth in the supplied medium, may have played a relevant role in the in sea-dominated coalesced communities, contributing to the species loss and the observed trend of declining biomass of primary producers (e.g. diatoms) in sea-dominated coalesced microcosms (i.e. RC_1:2_/OC_1:2_), while elevating the abundance of chytrids (parasitic zoosporic fungi). The abundance of bacterial cells in the sea medium supply increased as a result of the lack of complete cell removal during medium preparation. This likely provided an additional food source for grazers such as ciliates and flagellates during medium refreshments. If we hypothesize that this bacterial growth relaxed competition among grazers such as ciliates, we would expect a decrease in negative cohesion and an increasing trend in abundance of Ciliophora taxa across treatments with sea dominance. This, however, was not the case. Instead, ciliates exhibited a significantly greater increase in OC treatment compared to RC treatments, which were exposed to medium exchange from the affected sea medium supply to a greater extent. Nevertheless, future studies would clearly benefit from the simultaneous investigation of both bacterial and microeukaryotic communities in coalescence experiments to reveal the particular effects of trophic interactions.

The high number of unique microeukaryotes in coalesced communities suggests and supports an earlier finding that rare microbial taxa emerge during mixing events (Rocca et al. 2020). Such phenomena are most likely attributed to the selective advantage of certain phenotypes of these microbes under the new coalesced conditions, as well as the earlier described potential decline of certain microbes that opens niches and supports the establishment of emerging taxa. This explanation is also in line with the increased community stability (i.e. elevated fraction of competitive interactions) observed in the environmental coalescence treatments.

### Coalescence influences community stability

Community stability can be inferred from numerous community properties (Shade et al. 2012). Here, we approached community stability from the point of community cohesion, a metric that estimates the connectivity of microbial communities that stemming from biotic associations (Herren and McMahon 2017). As the authors highlight, taxa associations arise from biotic interactions and environmental drivers. Since the results of environmental coalescence treatments suggest low level of species loss (i.e. only 2%–8%), we may assume a strong support for competitive interactions alone when negative cohesion emerged. Positive cohesion can be indicative of both facilitative interactions and environmental synchrony, and these two cannot be disentangled in our present study. Nevertheless, the ratio of negative and positive cohesion allowed us to infer community stability of our observed communities, given that the cohesion values are indicative of negative- and positive-feedback loops, promoting or reducing community stability, respectively (Mitri and Richard Foster 2013, Coyte et al. 2015, Herren and McMahon 2017).

Although our findings suggest greater overall dominance of facilitative interactions and/or the influence of environmental synchrony (that is, the dominance of positive cohesion) across treatments, such dominance was limited by coalescence treatments, elevating the importance of competitive interactions that tend to be more evident in microbial communities (Foster and Bell 2012). The weakened dominance of facilitative interactions could potentially be attributed to disappearing reciprocal benefits (e.g. metabolic cross-feeding), since spatial structuring, that has similar effect, are unlikely in our microcosms (Harcombe 2010). In such scenario, the importance of ecological coselection, a phenomenon which aids members of a community to recruit one another, can be diminished and dominant taxa could not invade another community on their own, successfully (Diaz-Colunga et al. 2022). Although this reasoning is experimentally not tested herein, the increased levels of the inverse Simpson’s index in repeatedly coalesced communities (see e.g. Supplementary Fig. S6) may suggest such a phenomenon as it indicates mechanisms that counteract dominance. This might also explain why numerous microeukaryotes, such as ciliates and parasitic fungi, could elevate their abundances, following coalescence. This and other processes generated by environmental coalescence could have provided avenues for the introduction of additional species and/or the emergence of rare taxa that triggered competition to a greater extent and by doing so, leading communities towards greater stability. The driving mechanism behind this, as introduced earlier, originates from the increased number of negative-feedback loops which dampen the destabilizing effect of facilitative interactions that would otherwise lead to species loss. Our diversity estimates can support this reasoning as taxa richness increased in the coalesced communities, particularly in those that experienced repeated coalescence events, adding further evidence for a species maximum of microeukaryotes in brackish conditions (Filker et al. 2019, Tee et al. 2021).

Overall, our findings that community coalescence in estuaries strengthens microeukaryotic community stability by increasing the amount of competitions supports the notion that dampening the proportion of positive-feedback loops leads to even greater stability in microbial communities (Coyte et al. 2015), but questions May’s (1972) and Coyte et al.’s (2015) work on the destabilizing effect of increasing species diversity. The ground truth, however, most probably lies in between, as species diversity has been found to increase overall ecosystem stability when diversity is low, and decrease it when it is high (Pennekamp et al. 2018).

The composition of the coalesced communities with greater mixing ratio of the sea parent community generated greater predictabilities, suggesting that the predictability of community coalescence outcomes is significantly constrained in asymmetrical coalescence and is influenced by the eventual dominance of one parent community. Nevertheless, this predictability can be further enhanced as the frequency of mixing increases (i.e. repeatedly colliding communities). Lechón-Alonso et al.’s (2021) simulation study suggested that communities experiencing repeated mixing events should gradually shift from competitive towards more facilitative communities, which we did not find support for in this 16-day long study. Instead, regardless of the frequency of coalescence events (i.e. one-time versus repeated), our coalesced communities became more competitive than their parent communities.

## Conclusion

Overall, the composition of coalesced communities and the fate of parent community members are greatly influenced by the mixing proportion of parent communities, and to a lesser extent the temporal dynamics of community coalescence (one-time versus regular exchange). Our finding that community coalescence increases microeukaryotic diversity and promotes stability should be tested on microbial communities originating from other climatic regions and estuary systems with greater differences in salinity between endmembers to determine how these results can be generalized across estuaries. Additionally, we believe that assessing the effects of community stability on coalescence outcomes presents an intriguing avenue for future research. Although the more stable parent community dominated the final assemblages, which might have led to the observed asymmetrical outcomes, our study, with its single pair of communities, does not provide definitive evidence to either support or reject the notion that community stability influences coalescence outcomes.

Understanding the outcomes of community coalescence and the fate of microbes in their mixed environment is essential to understand and model biodiversity and associated functionality. A changed climate or influence of pollutants could modify coalescence outcomes (Vass et al. 2021, 2024), and even variation in weather conditions trigger more frequent and intense mixing scenarios (e.g. flooding and soil runoff into streams/rivers in response to heavy rainfalls) (Mansour et al. 2018). These processes will inevitably impact all features of estuarine ecosystems, including diversity, composition, function, and its capability to respond to various disturbances (Rocca et al. 2021).

## Supplementary Material

fiae100_Supplemental_Files

## Data Availability

Sequencing data is deposited to NCBI SRA database under the accession number PRJNA922225. Data (OTU table, consensus taxonomy and metadata) are available in Open Science Framework (OSF) (http://osf.io/sme36).
