# Supplementary material for: Microeukaryote community coalescence strengthens community stability and elevates diversity
Source: FEMS Microbiol Ecol. 2024 Jul 13;100(8):fiae100. doi: 10.1093/femsec/fiae100 (PMC11287207; doi:10.1093/femsec/fiae100)
Supplement: fiae100_Supplemental_Files [file fiae100_supplemental_files.zip › Supplementary data.pdf]

Supplementary Data

Microeukaryote community coalescence strengthens community stability and elevate diversity

Máté Vass[1,2*], Anna Székely[3], Ulla Carlsson-Graner[1], Johan Wikner[1,4], Agneta Andersson[1,4]

[1]Department of Ecology and Environmental Science, Umeå University, SE-901 87, Umeå, Sweden
[2]Division of Systems and Synthetic Biology, Department of Life Sciences, Science for Life Laboratory, Chalmers University of Technology, SE-412 96, Gothenburg, Sweden
[3]Department of Aquatic Sciences and Assessment; Division of Microbial Ecology, Swedish University of Agricultural Sciences, SE-750 07, Uppsala, Sweden
[4]Umeå Marine Sciences Centre, Umeå University, SE-905 71, Hörnefors, Sweden

*corresponding author's current address: mate.vass@chalmers.se, Division of Systems and Synthetic Biology, Department of Life Sciences, Science for Life Laboratory, Chalmers University of Technology, SE-412 96, Gothenburg, Sweden

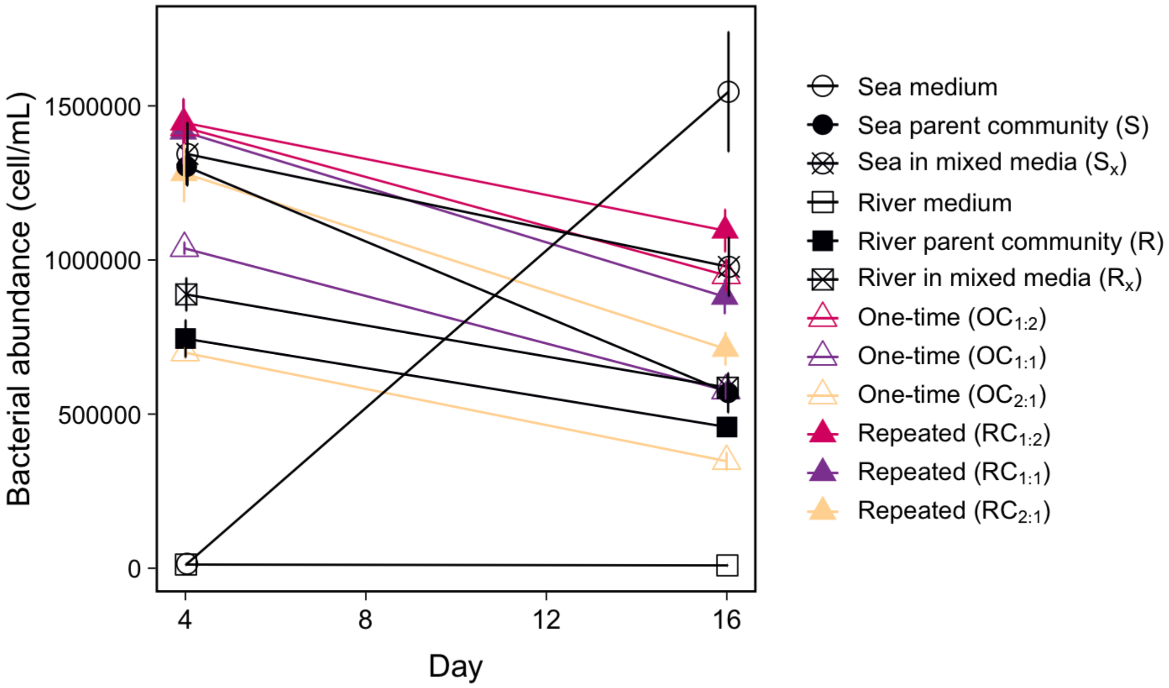

Figure S1. Bacterial cell counts in the treatments measured by flow cytometry.

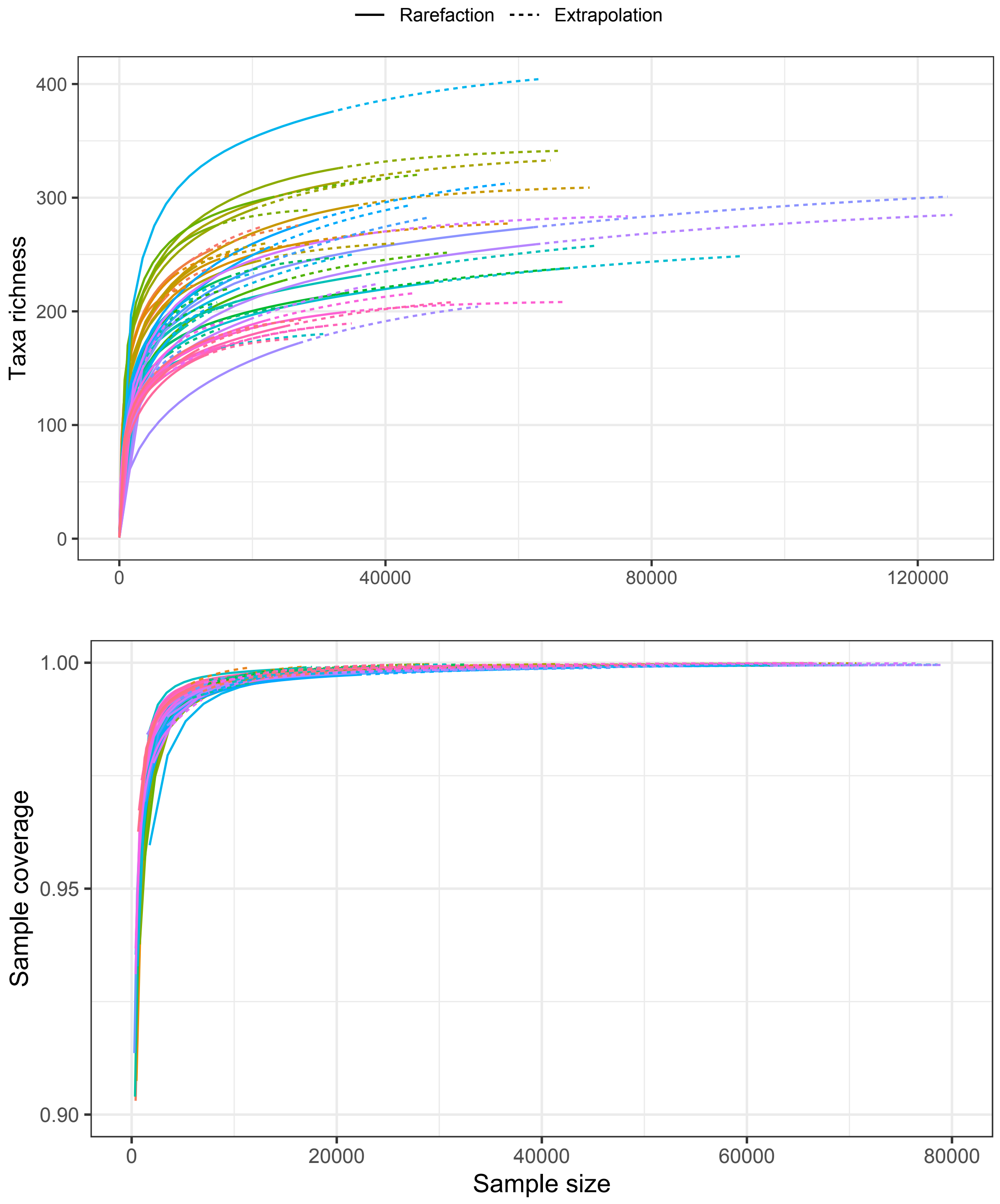

Figure S2. Diversity estimates and sample completeness (coverage) curves. Colours indicate different samples.

Equation S1: Conservative mixing model

We calculated the proportion of OTUs in the expected coalesced communities (ECC) based on the OTU proportions in the two parent communities (R and S) and the applied mixing ratios (i.e., 1:1, 1:2 or 2:1):

$$ECC = \sum_{i=1}^{n}(RA_{R,i} * MR_R + RA_{S,i} * MR_S)$$

Where i is an OTU, n is the total number of OTUs in the communities, $RA_{R,i}$ and $RA_{S,i}$ are the relative abundance of i OTU in parent community R and S, respectively, and MR_R and MR_S are the mixing ratio of R and S, respectively.

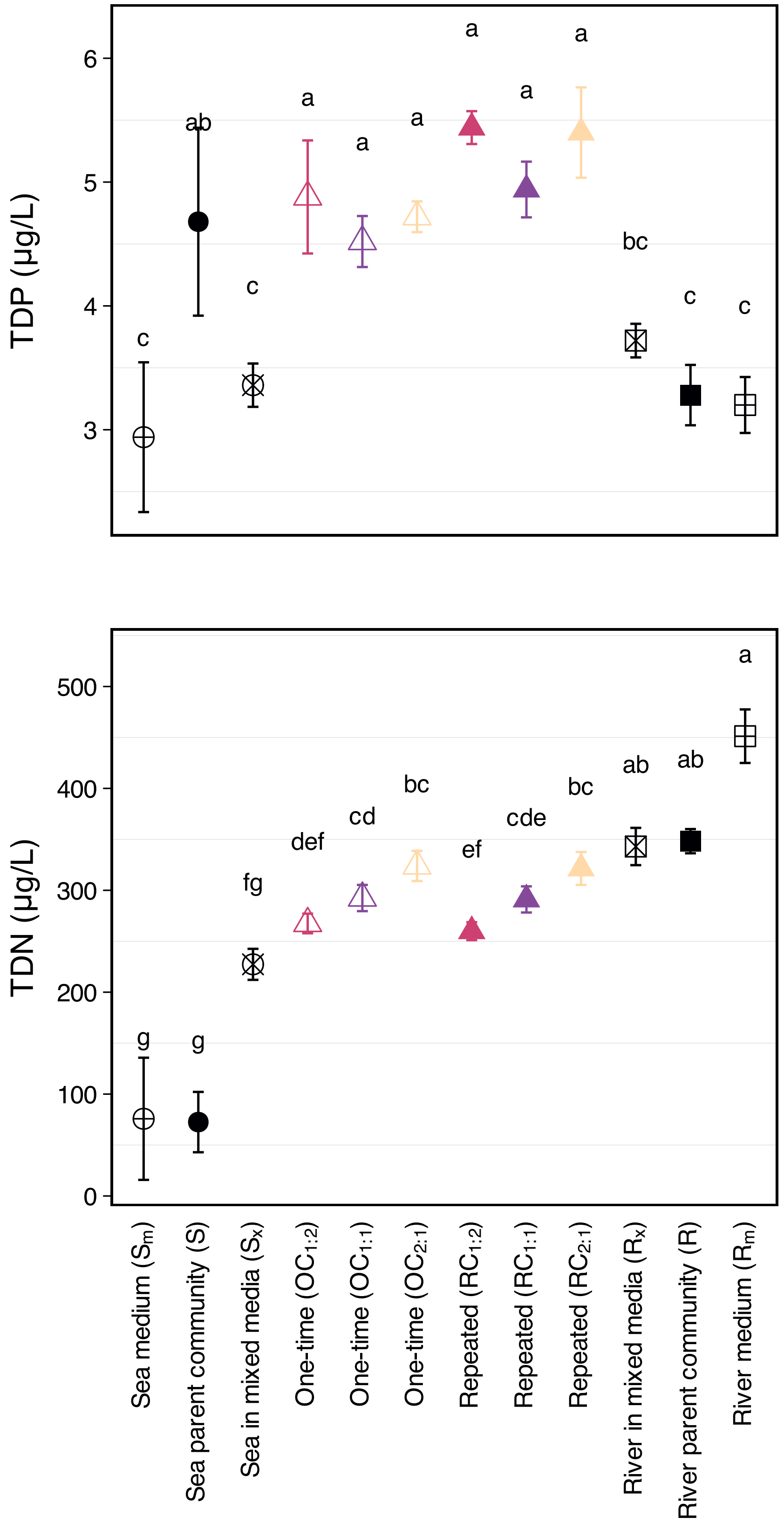

Figure S3. Differences in total dissolved phosphorus (TDP) and total dissolved nitrogen (TDN) across inoculum sources and treatments. N = 5 for each type of treatment. Significant (Kruskal-Wallis; $p_{adj} < 0.05$) differences are represented by lowercase letters. Error bars indicate standard deviations, and parentheses represent the mixing ratio of river:sea of coalesced communities.

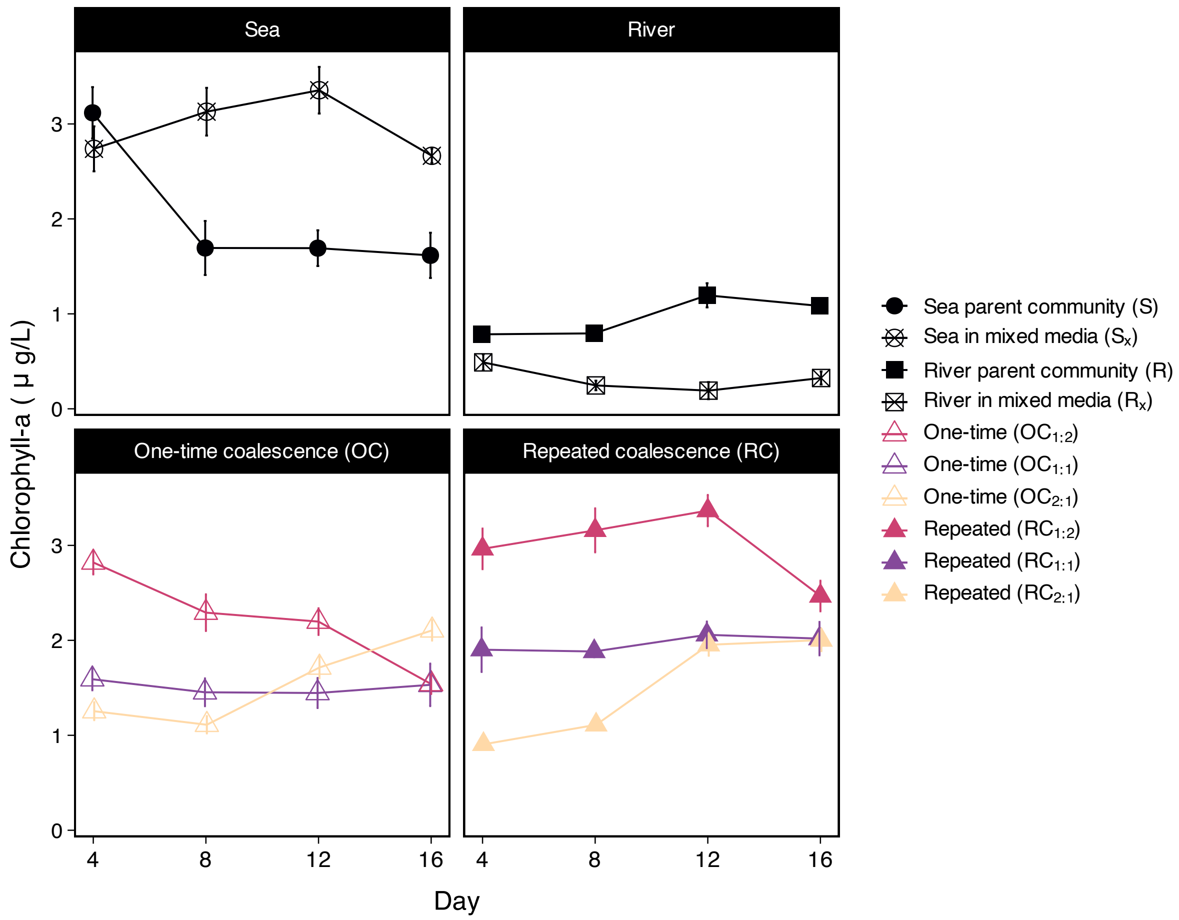

Figure S4. Changes in chlorophyll-a (as an estimator of the biomass of primary producers) as estimated by measuring the chlorophyll fluorescence-induced dynamic curve using AquaPen-C device (Photon Systems Instruments, Brno, Czechia). N = 5 for each type of culture and treatment at each date. Error bars indicate standard deviations, and parentheses represent the mixing ratio of river:sea of coalesced communities.

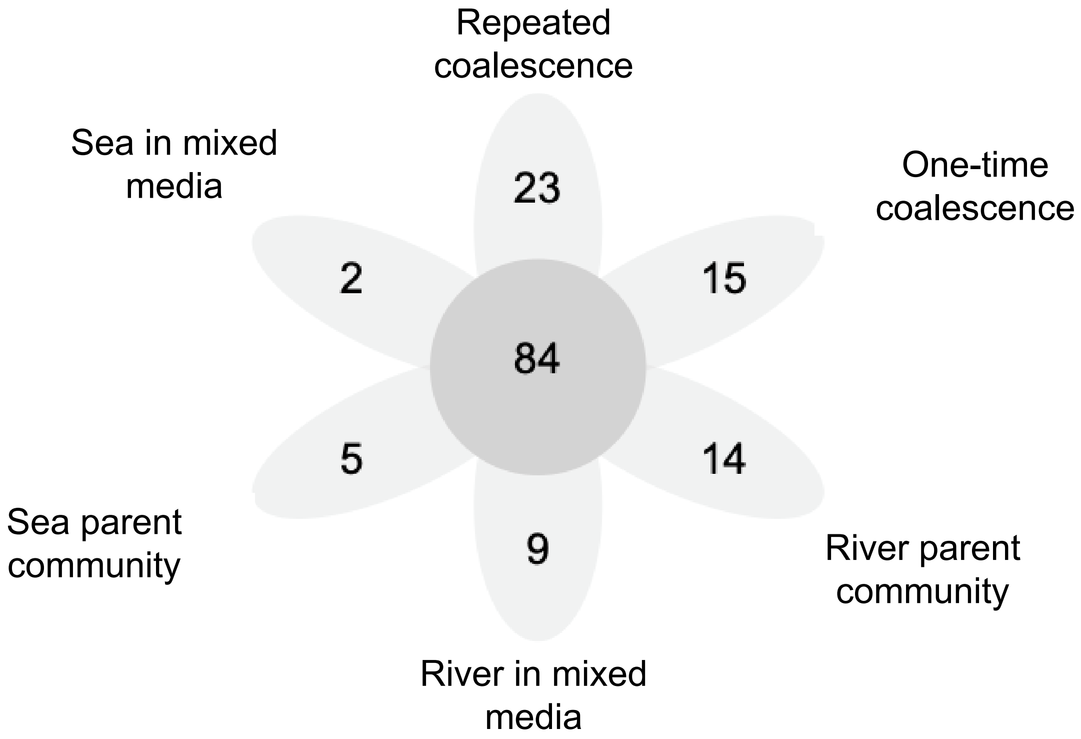

Figure S5. Number of unique and shared OTUs in the different inoculum sources and treatments. N = 5 for each type of sample and treatment.

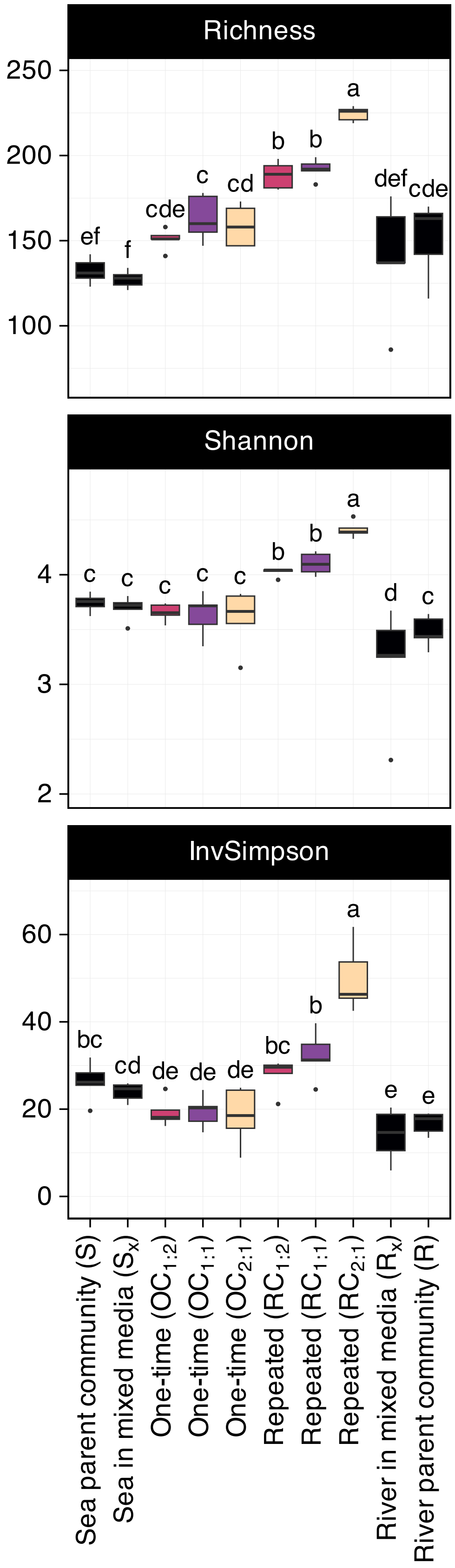

Figure S6. Alpha diversity estimates across samples and treatments. Significant post-hoc groups (Duncan's multiple range test for ANOVA; $p < 0.05$) are represented by lowercase letters. N = 5 for each type of each treatment. Error bars indicate standard deviations, and the mixing ratios of one-time and repeated treatments are indicated by the ratio of river:sea.

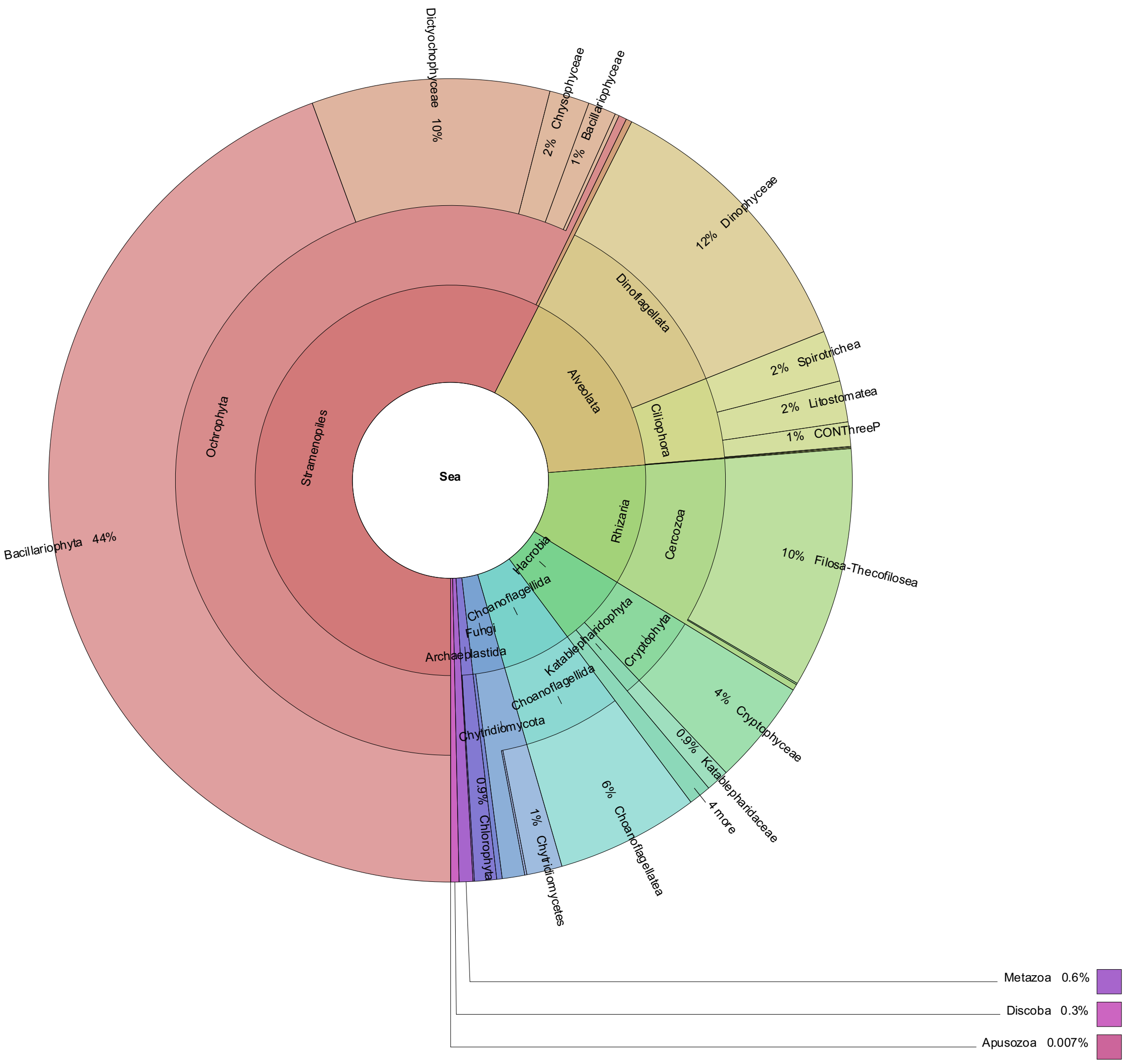

Figure S7. Taxonomic distributions of microeukaryotes, indicated as percentages of the total number of reads, found (day 16) in the sea inoculum microcosms (N = 5).

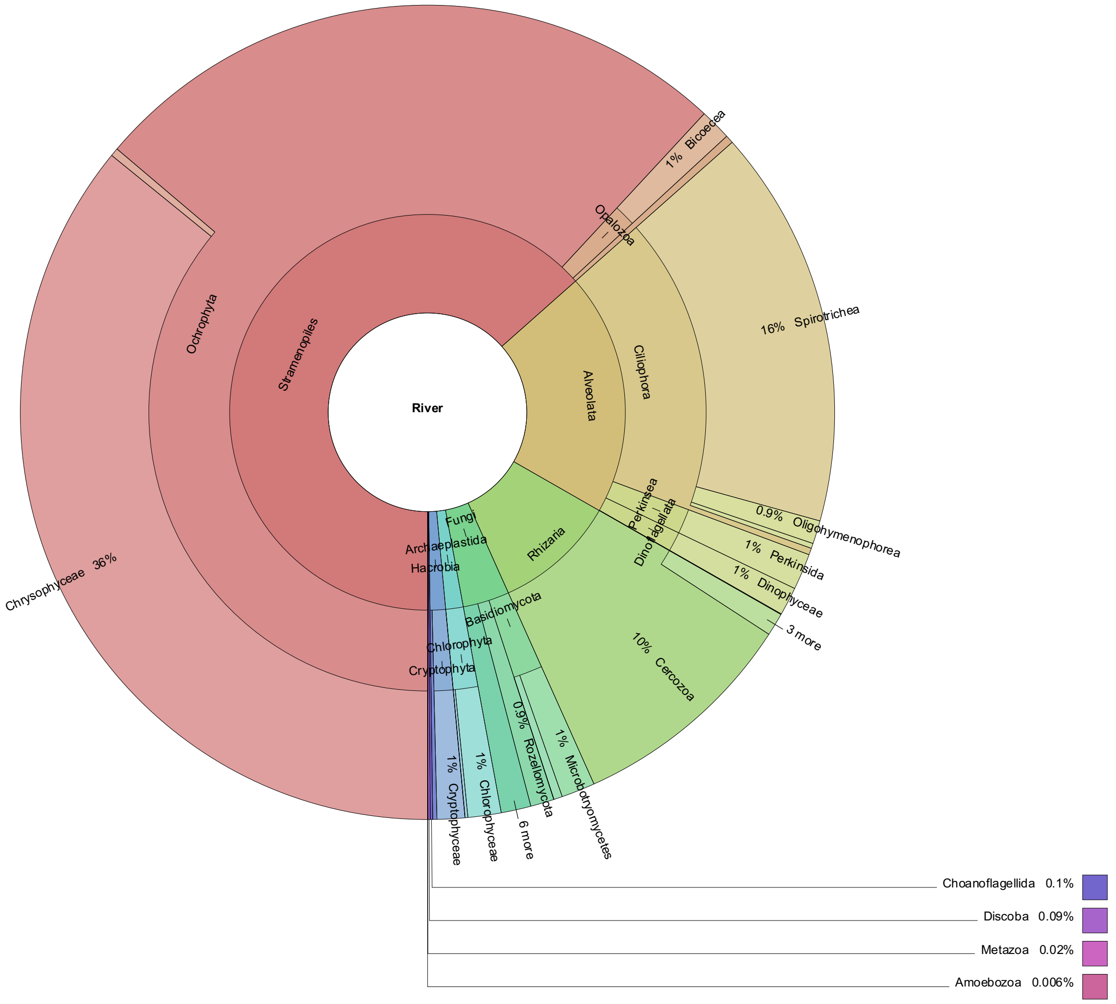

Figure S8. Taxonomic distributions of microeukaryotes, indicated as percentages of the total number of reads, found (day 16) in the river inoculum microcosms (N = 5).

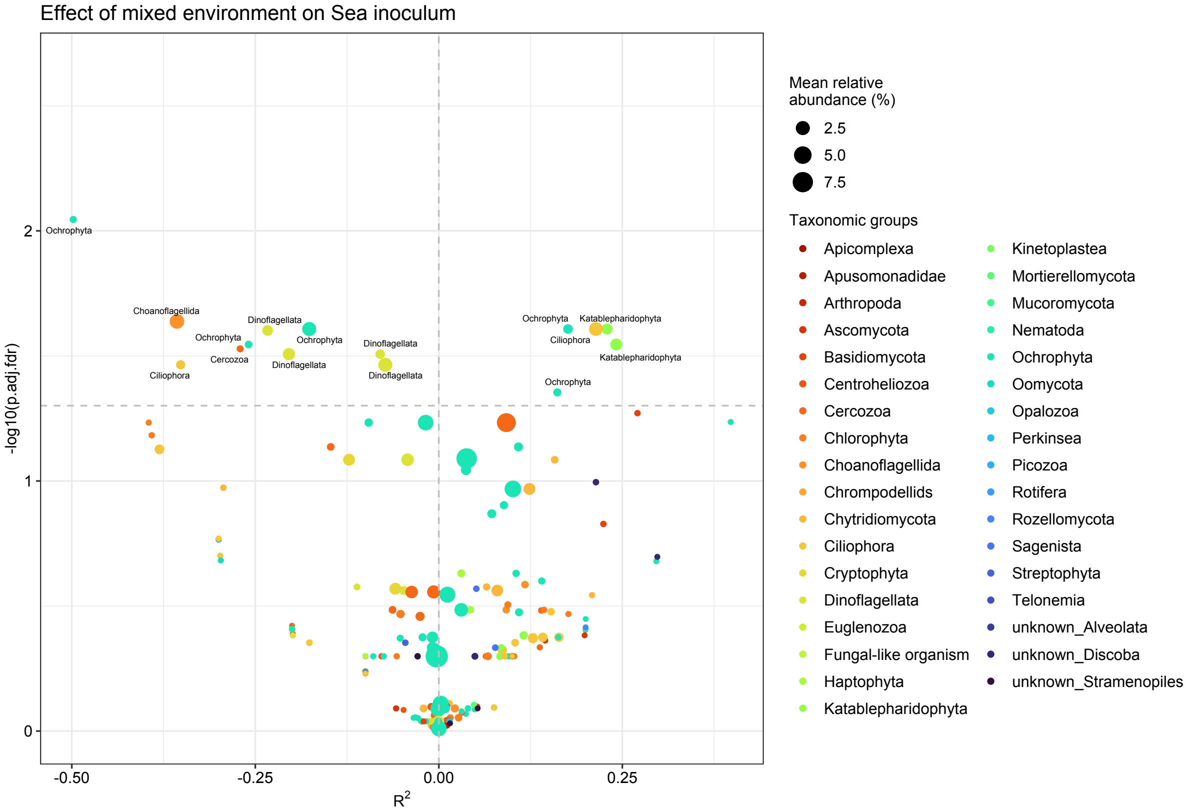

Figure S9. Differential abundance analyses of microeukaryotes of sea parent community after being incubated in the mixed environment (1:1 mixture of sea and river medium). OTUs above the horizontal dashed line indicate significant (*p* < 0.05; permutation-based FDR-adjusted p-values) increase ($R^2 > 0$) or decrease ($R^2 < 0$) in taxa abundance as assessed by ZicoSeq (Yang & Chen 2022).

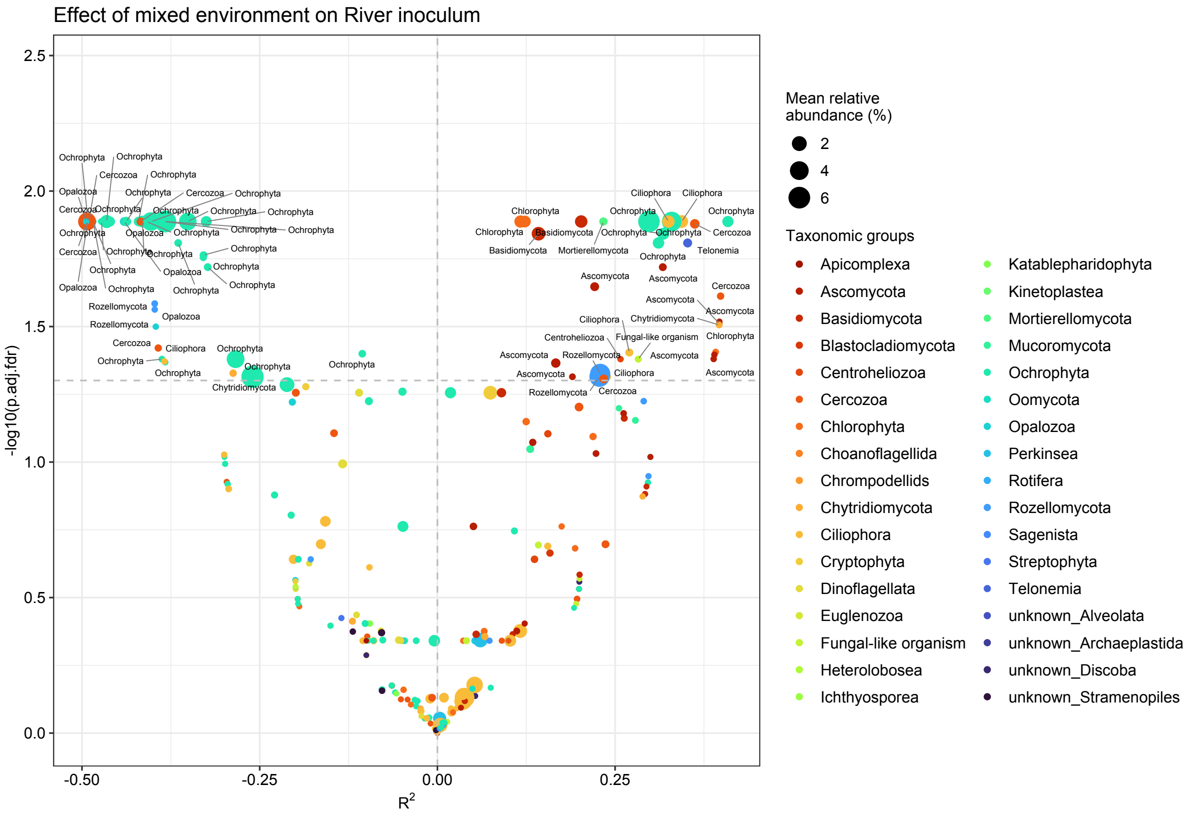

Figure S10. Differential abundance analyses of microeukaryotes of river parent community after being incubated in the mixed environment (1:1 mixture of sea and river medium). OTUs above the horizontal dashed line indicate significant ($p < 0.05$; permutation-based FDR-adjusted p-values) increase ($R^2 > 0$) or decrease ($R^2 < 0$) in taxa abundance as assessed by ZicoSeq (Yang & Chen 2022).
